# ColaborAtiva: A Platform Based on Gamified Collaborative Practices to Prevent Pressure Injuries for Wheelchair Users

**DOI:** 10.3390/s22051738

**Published:** 2022-02-23

**Authors:** Romero Mendes Freire de Moura Júnior, Lynn Rosalina Gama Alves, Carina Soledad González González

**Affiliations:** 1 Instituto Federal de Educação, Ciência e Tecnologia Baiano, Rua do Rouxinol, 115, Imbuí, Salvador 41720-052, BA, Brazil; 2 Instituto de Humanidades, Artes e Ciências Professor Milton Santos, Universidade Federal da Bahia, Rua Barão de Jeremoabo, s/nº—Ondina, Salvador 40170-115, BA, Brazil; lynn@ufba.br; 3 Ingeniería Informática y de Sistemas, Universidad de La Laguna, Calle Padre Herrera, s/nº, 38200 Santa Cruz de Tenerife, Spain; cjgonza@ull.edu.es

**Keywords:** collaborative practices, gamification, pressure injury, wheelchair users, social network analysis, platform, SUS, UEQ, NPS, ColaborAtiva, m-health

## Abstract

Pressure injuries are wounds caused by reduced blood circulation for regular and repetitive periods when pressing the human body against a hard surface. It is a pathology that needs daily preventive care and health promotion to avoid incidences. Thus, this article aims to validate a platform based on the gamified collaborative practices model to prevent pressure injuries. The methodological contribution is Design Science, and the software was evaluated by 16 wheelchair users who aimed at usability (SUS), user experience (UEQ), and ability to promote the system (NPS). In addition to creating a collaborative network using the interactions that occurred during the platform’s use. Wheelchair users rated 73.28 for SUS; on the UEQ benchmark scales, they classified: excellent (efficiency, dependability, stimulation, and novelty), good (attractiveness), and above-average (perspicuity) and in NPS obtained 62.5%. Moreover, they provided feedback to improve and legitimize that gamification positively interfered in the execution of collaborative practices. In the end, it is possible to suppose that the prototype contributes to the prevention of pressure injuries. In addition, it is possible to adapt it to apply to other pathologies that require continuous health care such as diabetes, mental illness, heart disease, addictions, multiple sclerosis, cancer, among others, maximizing its purpose.

## 1. Introduction

In a hospital bed or while using a wheelchair, people with reduced mobility are at risk of acquiring pressure injuries (PI), which are wounds that appear on people’s skin due to constant compression of the tissue by bone and a hard surface, such as a bed or a wheelchair [1]. However, several factors influence this pathology, such as poor nutrition, urinary and fecal incontinence, immobility, inadequate skincare, spinal cord injuries that cause insensitivity, low pressure, inadequate change of decubitus, friction, shear, and people with the aging of the epithelial tissue due to advanced age [2,3,4,5,6].

In Brazil, after searching scientific bases: Capes Journals, Scielo, PubMed, IEEE Xplore, BDTD, Scopus, and government documents, we did not find relevant data, at the national level, that reveal the number of expenses with PI and the number of people in these conditions. However, in Australia, research indicates that the treatment of PI is costly for the public health system, for the patients and their families. For example, Nguyen, Chaboyer, and Whitty [7] reported the annual expenditure of 983 million Australian dollars with this pathology in hospitals in Australia. The reason for this amount is due to the long treatment occupying time in hospital beds, the cost of materials for dressings and the high frequency of changing bandages. Moreover, depending on the wound’s degree of severity, drugs administration and even surgery are sometimes necessary [4,8,9].

Considering this context, this paper aims to validate a collaborative gamified platform to prevent PI based on the gamified collaborative practices model to prevent PI. This model was presented in a book chapter written in Brazilian Portuguese, called ‘Preventing pressure injuries’ [10].

Initially, we searched for correlated works, which brought studies on gamified collaborative practices on scientific bases, as can be seen on Table 1.

The results presented two documents related to gamified collaborative practices: the first one, called “A flexible customizable virtual escape room approach for interprofessional learners” [11] used an escape room as a gamification strategy to stimulate collaborative practices among students. Positive results were obtained with the interactions that took place both environments: face-to-face and remote. The experiment showed that collaborative practices and gamification, in this context, can increase involvement and collaboration between the participants. The second one, called “Improving the interprofessional practice, knowledge, and skills of health professions students through an interactive course in gerontology” [12], used gamification as a teaching technique in a team-based exercise for training to refine skills through collaborative practices for healthcare students.

The two documents demonstrate that collaborative practices when gamified bring benefits by offering new ways of interacting, stimulating their participants to collaborate in a playful and pleasurable way. However, there are still few scientific works on this topic, it is important to dedicate efforts to applying gamification in areas where the collaborative practices concept is already consolidated, such as health, education, law, and technology.

Gamification is not a game but rather a concept centered on the game elements’ application in different scenarios that are not games [13,14]. Although the gamification process has found several limits [15], its application in e-learning or health has demonstrated positive benefits [16,17,18,19,20].

Collaborative practices are present in several areas such as law, education, health, and computer science, where each of these fields has developed concepts based on the subjects and artifacts concerned and their relationships. Therefore, it is necessary to understand the term collaboration, which comes from the Latin *colaborare*, where the prefix (co-) is translated “as together” or “with” and *laborare* means “work.” Hence, it means collaboration is work done together.

Leifer and Meinel [21] infer that collaborating is to build something together, be it conceptual or physical, in an environment where everyone has the same intervention’s capacity and participation, with the possibility of leadership, however, without harming the conception of an egalitarian association.

Therefore, the World Health Organization (WHO) states that collaborative practice is the ability of professionals with different profiles to work together providing high-quality community care, directly associated with Interprofessional Education (IPE) which trains health workers to be part of a multidisciplinary team that, through collaborative practices, achieves common goals [22].

The collaboration’s concept is present in many applications, especially gamified ones. Such applications are characterized by using games’ logic in non-gaming environments, aiming to engage users in the platforms in a playful way and motivate them to adopt practices that are in tune with the platforms’ goals.

Therefore, collaborative practices are understood as actions where interaction occurs, aided in this process by artifacts that mediate the act. For example, when reflecting on a subject in a discussion forum, the action is to write, exchange opinions, points of view, and arguments using artifacts such as software and written language. Thus, the action becomes a collaborative practice that allows people to exchange experiences and discuss what was presented, creating a process rich in information that can directly or indirectly interfere in the already consolidated conceptual individuals’ structures.

An adherent example to the proposal presented here is the intelligent wheelchair simulator (IWS) in a virtual environment using an electroencephalogram (EEG) to capture brain waves signals and measure it associating the movement of the arms and legs, that is, which should think about moving the left fist and effectuating said act, noting the value measured by the EEG and repeating the process several times. Then, the procedure for the right fist, two fists, and the two feet is reproduced.

After that, they associated the frequencies’ pattern of each thought with the IWS shift commands: left, right, forward, and stop, respectively. Thus, they used game techniques in the simulator, asking the user to perform a command moving the IWS using a thought-generating trained frequency. The goal was achieved after seeing the displacement of the chair in the virtual environment [23].

This article is organized as follows: an introduction contextualizing the subject, and justifying and presenting the research questions, then materials and methods showing the methodological approach applied. The research instruments’ results display the findings from the methodological application path. The discussions answers the research questions based on results, and the conclusion synthesizes the subject and research contributions.

The main question that motivated this research was: (RQ1) In which way may a gamified collaborative practices platform assist wheelchair users in preventing pressure injuries?

To answer this question, we chose to use four perspectives: the platform’s usability, the user experience, the platform promotion’s degree by the user, and the gamification’s introduction in this system, that is, the environment is easy to use, the user felt good using the tool to the point of positively publicizing it and how the evaluators perceived the gamification. These scenarios generated the following questions: (RQ2) What is the level of the platform’s usability? (RQ3) Is the user’s experience positive? (RQ4) How much are evaluators promoting the platform? (RQ5) Does gamification influence the realization of collaborative practices?

## 2. Materials and Methods

Design Science (DS) used the methodological basis, which recommends artifacts’ creation to solve outlined problems, ensuring scientific rigor and practical research. Barbosa and Bax [24] (p. 34) said that “[…] many types of research prescribe artifacts as models and information systems […] where the most classic methodologies have limited scope.” Vaishnav and Kuechler [25] infer that DS conceptualizes absent knowledge using design, analysis, reflection, and abstraction.

**Figure 1:**
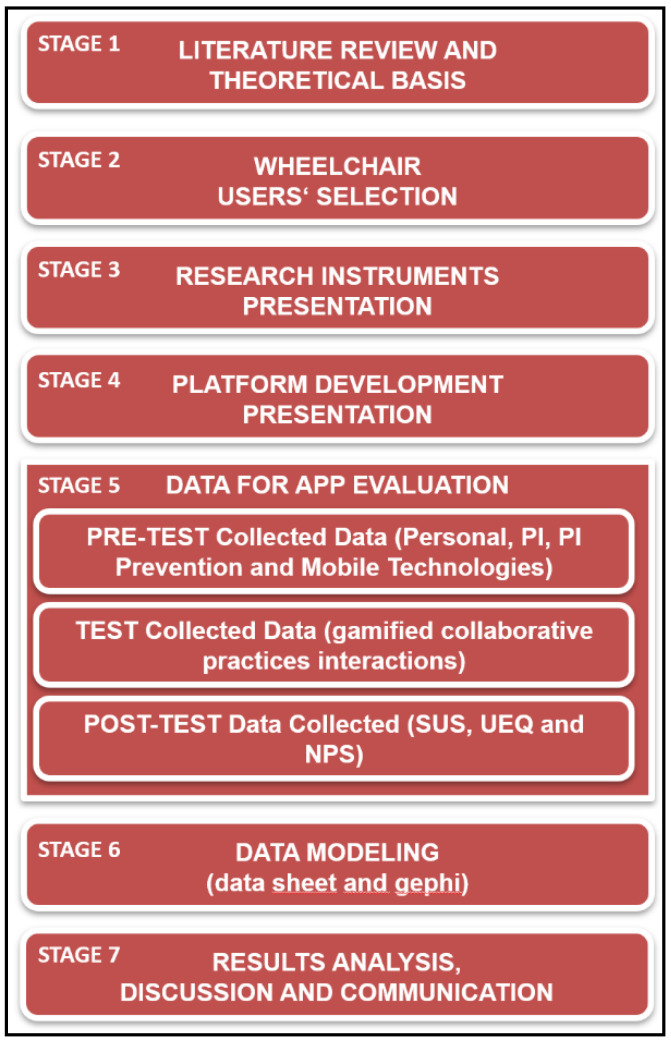
Research stages.

Figure 1 shows the research stages: stage 1 is presented in Section 1, stages 2, 3, and 4 are detailed in Section 2, and stages 5, 6, and 7 are outlined in Section 4.

The empirical space was the virtual community “Cadeirantes do Brasil,” (“Cadeirantes do Brasil” Facebook Group: <https://www.facebook.com/groups/490402347666154/>, accessed on 14 December 2021) on Facebook, created on 4 December 2012, with 16,200 members and as goals, “[…] questions, photo publications, videos and texts on a wide variety of topics”.

We posted an invitation calling for wheelchair users, adults aged 18 years or older with preserved cognitive functions, and smartphone owners with internet connections. We chose not to work with children and teenagers because adults are often embarrassed and ashamed when they are in an environment where young people master knowledge. Thus, they tend to retract and abandon the space that causes this sensation [26]. Preserved cognitive functions are necessary for the person to have the opportunity to interact and coexist with their peers using the existing technological resources with or without the caregiver’s help accessing the smartphone.

For this research, it was defined that data for analysis would be extracted using questionnaires and the platform’s database. Before using the platform to take information about personal data, the incidence of PI and access to mobile digital technologies were answered.

This information was essential to outline the evaluator’s profile, making it possible to make inferences about the results presented. The questions related to personal data (name, gender, age, and smartphone brand/model); pressure ulcers (Have you ever developed PI? How were your PI? Were you hospitalized? How long did you treat PI? Have there been any recurrences?); PI prevention (asking the volunteer individually if he/she/they performed the existing prevention items at platform check-in with answers in: always, often, sometimes, rarely or never. What are the reasons that make it challenging to perform PI prevention activities? Can people can influence PI prevention activities?); and mobile technologies (How much time do you use the smartphone per day? What smartphone’s functionalities do you use? Have you ever used a PI prevention app?)

The next step was the platform’s use for 7 days, and later, the participant filled the System Usability Scale (SUS) [27], User Experience Questionnaire (UEQ) [28], and Net Promoter Score (NPS) [29] instruments.

The SUS consists of 10 items:

1. I think that I would like to use this system frequently.
2. I found the system unnecessarily complex.
3. I thought the system was easy to use.
4. I think that I would need the support of a technical person to use this system.
5. I found the various functions in this system were well integrated.
6. I thought there was too much inconsistency in this system.
7. I would imagine that most people would learn to use this system very quickly.
8. I found the system very cumbersome to use.
9. I felt very confident using the system.
10. I needed to learn a lot of things before I could get going with this system.

This allows to verify the application’s usability considering positive questions: 1st, 3rd, 5th, 7th, and 9th and negative questions: 2nd, 4th, 6th, 8th, and 10th. For the first group, the answers varied from 0 to 4 points, subtracting 1 for each result obtained. For the questions in the second group, the result must be removed from 5; after that, the sum of all the answers is multiplied by 2.5, and the final result is classified in percentile on three groups [27]: for acceptability: Not acceptable (0–49); Marginal (50–69); Acceptable (70–100); by grade in the American grading system: F (0–59); D (60–69); C (70–79); B (80–89); A (90–99); and by the concept: Worst Imaginable (0–24); Poor (25–51); OK (52–72); Good (73–84); Excellent (85–99); Best Imaginable (100).

It is important to mention that SUS achieves between 90% and 100% results’ accuracy, according to Tullis and Stetson [30], based on 12 people’s assessment, that is, it is not essential to carry out tests with large groups to obtain precision results. Nielsen [31], in turn, ratifies that those 15 participants allow us to identify about 90% of the existing problems.

The UEQ has 26 items divided into six scales: attractiveness, perspicuity, efficiency, dependability, stimulation, and novelty. Each question refers, for example, to an antagonistic pair: unpleasant/pleasant, fast/slow, or conservative/innovative and should be measured following a linear scale with seven elements, as seen in Table 2 [32], when evaluating the original/conventional concept, the numbers from 1 to 3 classify the product as original, from 5 to 7 as traditional, and the number 4 is neutral. Thus, the idea of proximity follows, the extremity is more intense; that is, 1 and 7 are considered very intense, and as they move towards the center, they become milder [28,29,30,31,32].

For analysis purposes, the UEQ treats each item on a scale from +3 to −3, with the positive concept receiving the highest value and following the sequence to the other margin. Thus, the questionnaire’s analysis is done by scale means, mean and standard deviation per item, confidence intervals, and distribution of the answers. This allows inferences about the user’s behavior and feelings about the evaluated product [28,32].

The NPS consists of one question: “On a scale between 0 and 10, how much would you recommend the ColaborAtiva platform to a friend?”. The answers are consolidated into three groups: from 0 to 6 are considered detractors who demonstrate dissatisfaction with what they are evaluating and usually spread this feeling with their neighbors; from 7 to 8 are the passives who feel taken care of but not excited about what they are judging; and from 9 to 10 are promoters who value the item above expectations and invite their peers to use it.

In the end, the NPS is calculated by subtracting detractors’ percentage from promoters’ percentage, classifying them as follows: Excellence’s Zone (76–100), Quality’s Zone (51–75), Improvement’s Zone (11–50), and Critical Zone (0–10). This score can be segmented by age, gender, education, educational level, economic activity, or social class to make inferences and look for behavior patterns. Thus, this indicator offers the chance to identify detractors and apply strategies to transform them into possible promoters [29].

Another instrument used was the data storage resulting from participants carrying out collaborative practices through interactions with the platform, which will help to elucidate the behavior and role of each individual in the platform’s dynamics. This information allows the graphs’ production where it is possible to identify the network’s degrees, its density, its diameter, its subgroups, its central elements, its centrality measures, modularity, and other items that permeate the complex networks’ universe [33,34].

**Figure 2:**
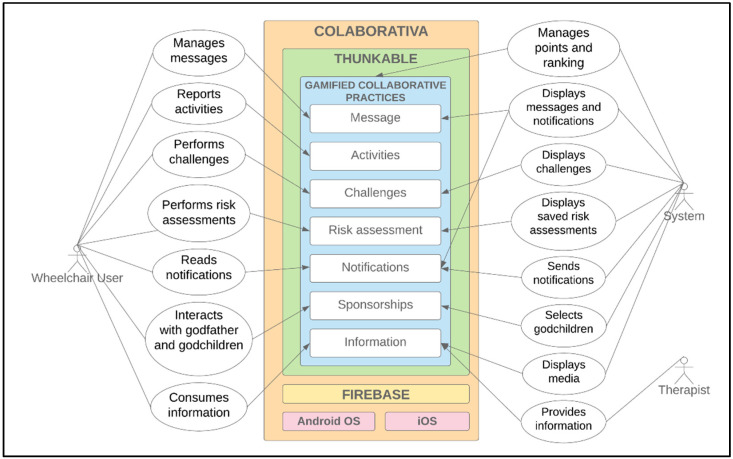
ColaborAtiva architecture.

ColaborAtiva platform has this name to highlight users’ need for active collaboration. It is a platform whose main objective is to carry out daily activities to prevent PI. Figure 2 shows ColaborAtiva’s architecture, where actors interact with the platform through gamified collaborative practices.

The platform has the interfaces presented and detailed in Table 3; all of them are gamified, through the collaborative practices’ execution and the goals’ achievement, where users score and are rewarded in an environment where the more you act and collaborate, the more you achieve highlights.

**Figure 3:**
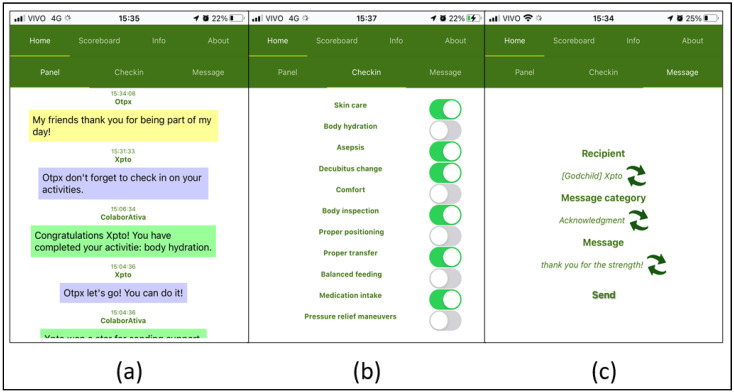
ColaborAtiva Screenshots. (**a**) Panel; (**b**) Checkin; and (**c**) Sending and Receiving Messages.

Figure 3 presents the main screenshots of ColaborAtiva. The current version of the platform is now available in Portuguese and English.

The platform was made available for Android and iOS since, according to Kantar [35]. In December 2021, they represented 92.8% and 7.0%, respectively, of the smartphone operating systems of Brazilians.

Despite demonstrating that Android OS dominates the Brazilian smartphone market and is a free project with accessible development tools. The desire to offer this platform to other countries and, since the Thunkable development platform offers the option to create applications for both Android and iOS, it was decided to implement them for both OS. Thus, it was possible to guarantee 99.8% of Brazilian smartphones with this choice.

The development tool used was Thunkable Cross (Thunkable Platform: <https://thunkable.com/#/>, accessed on 14 December 2021) which generates software for Android and iOS devices. In addition, it does not limit the number of screens used; the application size is up to 50 Megabytes (MB), it has limited documentation, but the community is very active, it has native integration with the Firebase (Firebase Platform: <https://firebase.google.com/>, accessed on 14 December 2021) database, which is based on JavaScript Object Notation (JSON), offers simple tests directly on smartphones.

However, there were difficulties in development that were circumvented but should be itemized for registration. For example, the development platform’s limitations are a lack of components, especially for manipulating dates to delimit the number of days, weeks, and months. Moreover, the constant updates of Thunkable Cross originated from errors in parts of the platform that we are working on and therefore, influenced the implementation time of the platform.

The data for evaluation were collected pre-test, during the test and post-test. On the first one, the participants, after answering the invitation post in Facebook’s group, “Cadeirantes do Brasil.”, got in touch and received details about the research and its stages, a general panel about the platform was created, and a questionnaire about participants’ profiles was given. After filling out the questionnaire, a step-by-step guide was sent to guide the smartphone installation.

The experimental phase took place with wheelchair users accessing the system for 7 days. Based on the availability of dates provided by the volunteers, this was the maximum possible period in which everyone could use the platform. Individual use would not allow us to observe the interactions in realizing gamified collaborative practices on the network.

In general, the participants used ColaborAtiva with no problems and without mediation by researchers. The interactions established by users were respectful, and they knew how to deal with situations arising from a group relationship. It is essential to point out that all collaborative practices and interactions were stored in the software’s database for analysis.

After completing the experiment, a questionnaire was sent containing questions about prevention activities, usability, user experience, and system promoting possibilities. Thus, the data from questionnaires were collected for modeling and analysis.

## 3. Results

The data organization took place in three stages: the information tabulation from the profile questionnaire; the SUS [27], UEQ [28], and NPS [29] instruments; and users’ interactions during the experiment. These interactions were modeled in graphs based on network theory and designed on Gephi (Gephi Software <https://gephi.org/>, accessed on 14 December 2021) software, version 0.9.2.

The data were prepared regarding considering the practices performed on the platform and stored in the database. First, we used the messages exchanged between users, converting them from JSON to comma separated format (CSV) files. The date and label columns were used to identify the day and practice performed, respectively.

Thus, the practices were consolidated into a single file with two columns: the first one, called TARGET, containing the system notification recipient of the message sender. Moreover, the second one, called SOURCE, is filled with the practice’s labels. The file was imported into Gephi, and the subjects’ names were changed using a sequential number starting from 01 preceded by the letter S to preserve the volunteer’s identity. The procedure was repeated for messages exchange between the participants, separating the columns into sender and recipient.

For this study, 16 wheelchair users volunteered; see Table 4. It is important to note that the low adherence to this scientific research is that although the group has many participants, not all are wheelchair users, and not all are active. In addition, scientific research in Brazil has free membership through volunteering without any remuneration or advantage. Another aspect is that there is no country culture and incentive to participate in scientific research.

The group who evaluated the platform was distributed by age range in decades between 20 and 59 years with a more significant presence of individuals between 30 and 39 age range totaling 44%, 62.5% of subjects who were female, and there was a representation of both genders in all age groups.

Thus, the participants owned smartphones from four manufacturers: Samsung, Motorola, LG, and Apple. Only the last one has iOS operating system [6%], while the others are based on Android technology [94%]. It should be noted that 15 evaluators experimented using the Android OS and 1 using the OS IOS.

Regarding all devices used, we had iPhone 8 Plus, LG K10 and K11, Motorola G8 Plus and Samsung A51, S10, J2, J5, J6, J6 Plus, J7, and J7 Prime-had processors with 4-core or 8-core, frequencies ranging from 1.2 GHz to 2.39 GHz, RAM from 1 GB to 8 GB and storage between 8 GB and 256 GB. Thus, no problems were reported to prevent the platform’s use, showing that it is possible to use it with various configurations from the simplest to the most robust, leading to set as a minimum configuration for ColaborAtiva: a 4-core processor, with 1.2 GHz of frequency, 1 GB RAM and storage of 8 GB.

The evaluators declared the amount of time devoted to using smartphones, and we separated them by gender and age group. We noticed that 15 (94%) people use smartphones for more than 1 h/day, 10 (63%) use them for more than 5 h/day, and only 1 (6%) subject declared to use the device for less than 1 h/day. Associated with this, mostly female volunteers (70%) reported they use their devices for more than 5 h/day. In contrast, male users equally divided their statements between 1 and 3 h/day (50%) and more than 5 h/day (50%).

Next, the result of the three instruments outlined in the methodology: the SUS that evaluated usability, UEQ the user experience, and the NPS the ability to indicate and disseminate the platform among people. Complementing this, complex networks were also used to represent and quantify the relationships between the subjects, the collaborative practices carried out, and the interactions between their peers.

The SUS was the first tool made available to evaluators; it was developed based on three pillars: efficiency, effectiveness, and satisfaction, but focused on the user’s perception more subjectively. In this context, it seeks to capture whether people reach their goals using the evaluated artifact, how much effort was expended, and how comfortable it was all the way to reaching their goals [27].

**Figure 4:**
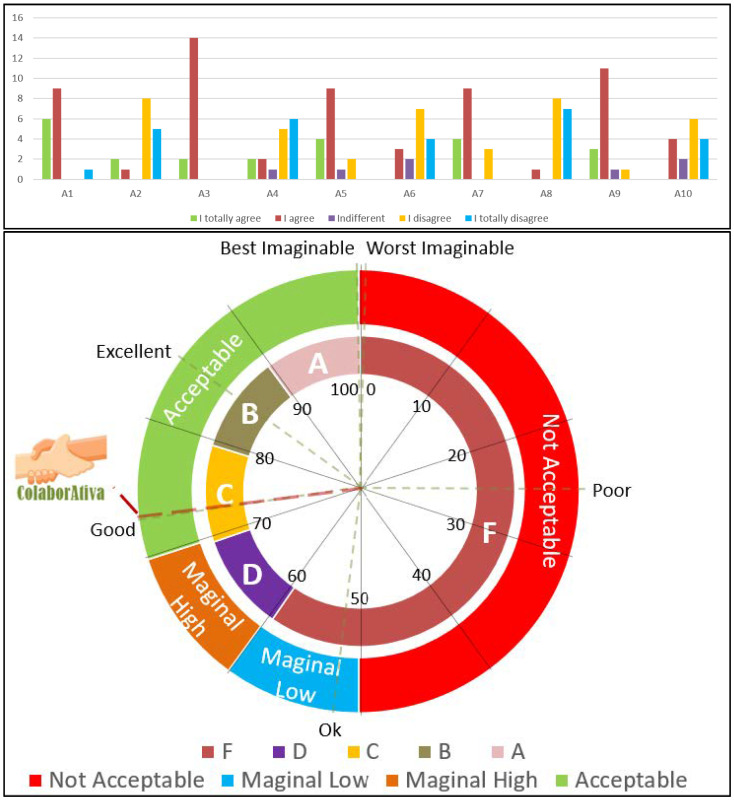
SUS answers and ColaborAtiva’s classification.

Figure 4 shows the platform’s usability level; when visualizing the SUS scale, it identifies usability through three categories: acceptability, degree, and concept, as already explained in the methodology. This occurs because the value between 0 and 100 presented is measured in percentiles for the respective categories, i.e., the number presented should not be considered a percentage or a rating score [27].

Thus, the volunteers evaluated the platform with an average score of 73.28, classifying it as acceptable, with a grade C in the American rating system, and considered conceptually sound.

**Figure 5:**
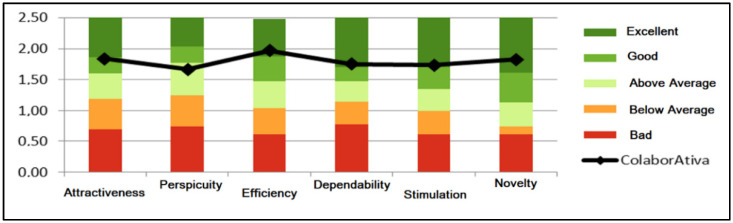
Benchmark graphic generated by data analysis tool provided by UEQ [28].

The following instrument made available was the UEQ that sought to evaluate the user experience about the experienced artifact. Figure 5 presents the benchmark available in the data analysis tool provided by UEQ developers.

The results of questions asked to the subjects were distributed into six categories: attractiveness, perspicuity, efficiency, dependability, stimulation, and novelty. The first indicates whether people like the product or not; the second manifests the ease of use and learnability in its handling; the third registers the speed and simplicity in performing tasks; the fourth point out the feeling of mastery over the tool; the fifth designates how motivating and fun the platform is; the sixth measures creativity in design and user interest [28].

**Figure 6:**
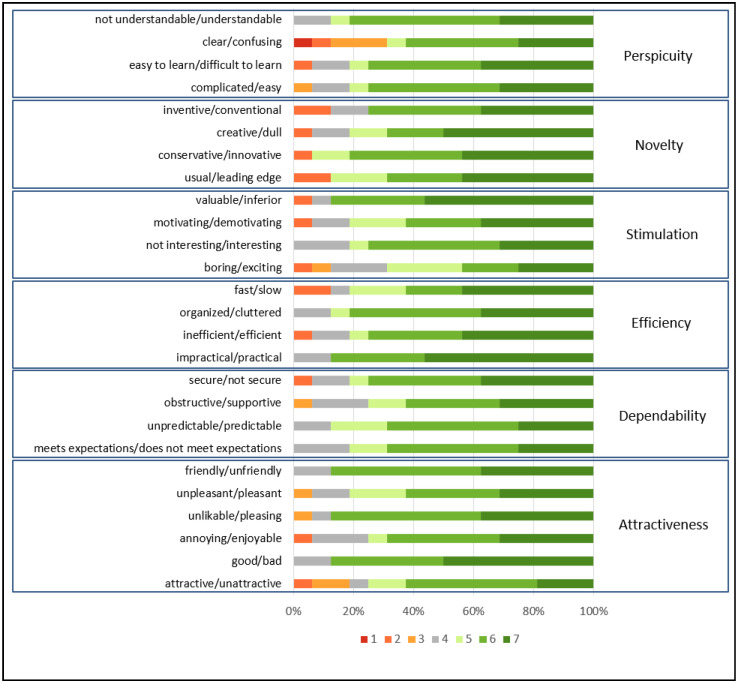
Answers’ distribution graphic generated by data analysis tool provided by UEQ grouped by scales [28].

Figure 6 presents the UEQ distribution board of each answer grouped by category.

The third instrument offered was the NPS, which, despite being composed of a single question, has its main objective of identifying problems in promoting the application. As a result, the NPS presented values containing: 75% promoters, i.e., 12 people informed that they would indicate the platform; with values 9 or 10, 2 passives (12.5%) who marked values 7 or 8; and 2 detractors (12.5%) who marked values lower than or equal to 6. In the end, the calculated NPS answers the third investigative question about the degree of promotion of the platform by the evaluators, which was 62.5% considering the result of the subtraction between the percentage of promoters and detractors.

**Figure 7:**
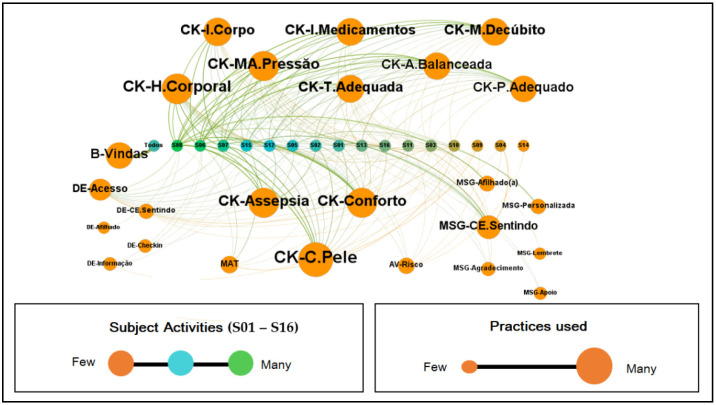
Graph of Subjects’ Interactions with ColaborAtiva.

The instruments presented above extracted the sensations of wheelchair users concerning usability, user experience, and promotability. However, the following graphs showed the effective behavior of the volunteers when using the platform, which allowed us to infer about their actions in the system, as shown in Figure 7.

Figure 7 is a graph modeled in Gephi, formed by the subjects’ interactions with the collaborative practices existing in the platform. S01 identified the participants to S16, and their colors indicate the intensity of their activity within the system. The sizes, in turn, designate proportionally the use of the application practices, i.e., the more the resource was used, the larger the text and the sphere presented in the graph.

Finally, the following challenges were met: accessing the application, sending messages about how I am feeling, completing 100% of the check-in, accessing the support material, sending messages to the sponsee that earned the challengers’ notifications on the board informing all participants of the achievement of the goals, stars on the challenge board, and points that were accumulated with the other practices carried out (Table 5).

Table 6 shows the observations of some participants who stated that the scoreboard and the challenges provided influenced them. Subject #1 felt excited when he saw his position improve; #2 strived to reach a good score; #3 felt motivated to do the activities; #4 was influenced to continue doing the activities; #5 noticed that he was among the first and that this affected his emotional state; #6 felt driven to compete with his peers and thus wanted to gain more points, and #7 wanted to do the practices to improve his position among his peers.

**Figure 8:**
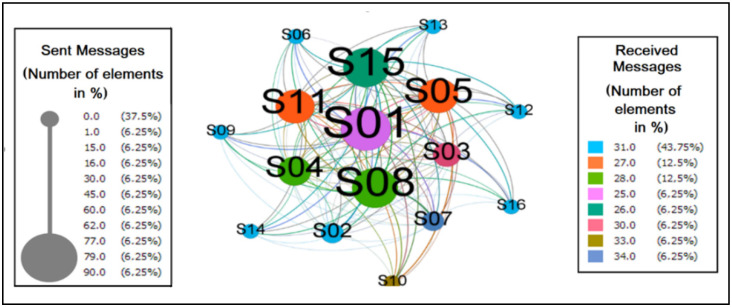
Messages between peers by weighted input and output grades.

Figure 8 represents the messages processed between the wheelchair users during the experiment. It is a directed graph with 16 vertices (participants) and 136 edges (relations), with the density of 0.567 indicating that approximately 57% of the possible connections between subjects were established.

Other measures that showed the cohesion of this network were related to the degree of connection, where the average degree is 8.5, the average weighted degree is 29.7. The average edge weight is 3.5, showing that each subject has eight connections and repeats each of them approximately four times. The weighted input degree showed that S07 and S10 received the most messages proportionally. However, the range of values for this metric was short, between 25 and 34, which indicated that the subjects generally received an equivalent number of messages with slight variations. On the other hand, in the weighted degree of output, the distance between the values was wider, ranging from 0 to 90, i.e., the numbers are more dispersed, which gives greater prominence to S01, S08, and S15 as those who sent more messages, proportionally, in the network.

**Figure 9:**
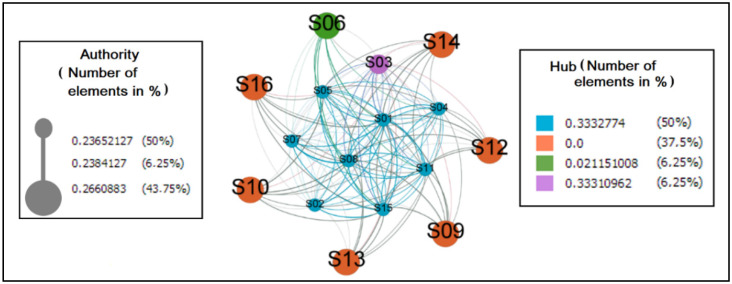
Messages between the HITS (authority and hub) pairs.

The graph in Figure 9 reflects the behavior of individuals when it comes to identifying them as authorities or hubs in the network. The first was valued by the number of messages received, amplified by the importance of the senders. On the other hand, the second had its value measured by the number of messages sent, amplified by the notoriety of the receivers.

As hubs and authorities, the quantity of eight and sever elements, respectively, were considered, where the former is arranged in the center and the latter at the margin. It was detected in this scenario that six elements did not perform the practice of sending messages. It is believed that it is necessary to study this phenomenon to find out why this is abstention. However, it is inferred that this value could be different by improving the messaging system so that participants receive notifications on their smartphones when actions occur in ColaborAtiva.

**Figure 10:**
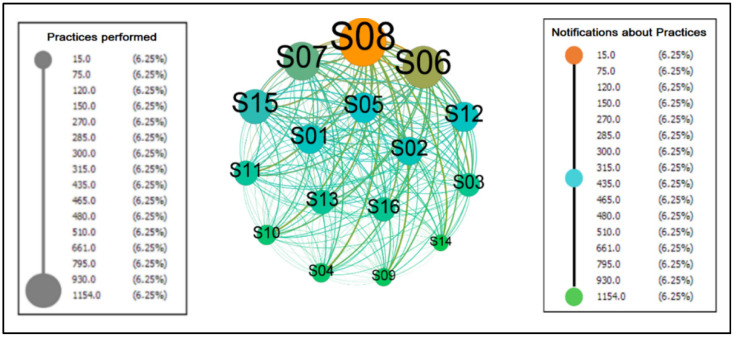
Collaborative Practices Carried Out in ColaborAtiva.

Figure 10 represents the collaborative practices performed by the subjects within the platform.

It is a directed graph, with 16 vertices and 240 edges, diameter, and density with a value of 1.00 indicating that 100% of the possible connections between the participants were established, that is, we have a complete graph. Furthermore, due to this condition, the metrics of centrality (intermediation [0.0], proximity [1.0] and eigenvector [1.0]), eccentricity [1.0], clustering coefficient [1.0] and HITS (authority [0.25] and hub [0.25]) reach the same values for each node creating a homogeneous environment where all are integrated among themselves, indicating that there is no differentiation of importance and prestige in the network, that is, it is a space in which the communication flow occurs without dependence on intermediaries as desired for a collaborative network.

The average degree, weighted average degree, and edge weight are 35, 435, and 29, respectively, indicating that each node has 29 connections. The weighted degree of entry and exit were the values that presented differentiation in the network. They were based on the weight of the edges, and these counted from the repetition of the communicational flow between the actors.

Table 7 presents the post-experimental observations made by the volunteers in the experimental evaluation.

## 4. Discussion

Regarding the representation of different groups of our sample, we can note that Brazil has continental dimensions, so even within the country, there is a plurality and cultural and usage diversity that did not bring divergent elements in this investigation but could have presented different biases.

According to our results on the amount of time devoted to smartphones, we found differences between female and male participants. Thus, the female audience attested that they stay connected longer than males; this information corroborates with Lee et al. [36], who studied the behavior of 1236 university students in South Korea and published that the percentage of smartphone use over 4 h for men was 29.4% and for women 54.0%. However, about the performance of daily practices and communication within the system, male volunteers performed an average of 26.8 practices and sent 30.5 messages instead of 26.4 and 29.2, respectively, of females.

Thus, it is not possible to state a direct relationship between age and time on mobile devices use. However, it is possible to understand a generation gap in smartphone use; for example, Statistics Canada [37] details the habits of different age groups. Table 8 presents discrepant differences between the percentages of the extreme ranges, i.e., the younger population included the use of the smartphone in their main routines. In contrast, the older ones remain distanced from the device. It is a fact that after a decade, the entire population of the first group will migrate to the next, and approximately 50% of the other groups will move to the next, i.e., the trend is that these percentages will increase. The smartphone will become increasingly consolidated in the human routine.

A factor that is contributing to the increase in the time of use of smartphones is isolation and social distancing declared by WHO [38], since 11 March 2020, due to the pandemic caused by the new coronavirus, which has lasted so far 2 years and, consequently, reducing the possibilities of face-to-face socialization. Thus, people have been compelled to enter or intensify the use of technologies to maintain interaction with the outside world. In this context, it is necessary to briefly approach digital literacy as an essential aspect in appropriating these processes. According to Almeida and Alves [39] (p. 7) digital literacy is a concept

[…] associated with social practices also influence cultures and people who do not master writing. In other words, digital literacy goes beyond the domain of techniques, skills, and abilities to use reading and writing on the screen. It becomes a broader process that operates in different spaces and contexts.

In this way, each person builds their knowledge according to their needs and the technological contributions they have, developing practices that transcend the technical, such as critically evaluating information from multiple sources, understanding the context, interpreting and communicating with different cultures respecting and understanding them. From this perspective, age is not a determining factor in learning and access to technologies, for what matters is the will and desire to reach new heights and position oneself in the digital world.

It is worth mentioning that ColaborAtiva was developed to include wheelchair users in general, regardless of how long they have had access to mobile devices, their gender, or their age.

About the usability of the platform, the results obtained with the SUS confirm the validity of ColaborAtiva regarding usability; that is, it is a software that people consider acceptable for what it proposes. However, the observations made should be considered in which the evaluators identified modifications in the platform to improve its usability. The most requested change was to send alerts on the smartphone containing notifications, messages, and reminders as observed by one of the users: “It could issue alerts reminding of activities or when a message is directed.” Another feature would be a greater dynamism in the messaging system bringing it closer to the existing models in instant messaging applications.

Related to the evaluation of the User Experience through the UEQ, we can note that according to Hinderks, Schrepp, and Thomaschewski [28], “Comparing the results of the evaluated product with the benchmark data allows conclusions about its relative quality compared to other products.” Thus, the results of ColaborAtiva compared to other products evaluated by UEQ show that concerning attractiveness (1.84), efficiency (1.97), dependability (1.75), stimulation (1.73), and novelty (1.83), the platform is among the 10% best results. Regarding perspicuity (1.67), it was situated in the range above the expected, that is, between the 25% best and above the 50% worst results.

Based on this research, it was found that more attention is needed regarding the ease of use of the platform and its learning. It becomes important to review the elements that promote the interface between the application/user. In new versions, mechanisms will be created to improve this relationship, allowing the user to know what the application offers and how they can take full advantage of it.

It is possible to realize, in Figure 6, that the clear/confusing option was the item that presented the highest number of negative values, which ratifies the care with perspicuity. However, it is noteworthy that when questioned about the negative item, users argued that they did not know what to do on the platform. However, none of them had read the folder created to explain how the platform works. Thus, new ways of interaction should be sought that can dialogue with the volunteers during the use of the software to allow greater synchrony between the doing and explaining how, why, and what to do.

The results obtained by the NPS showed that ColaborAtiva is in the Quality Zone (51–75), which represents a platform that is recommended, but that still presents items to be improved in order to reach the Excellence Zone (76–100).

Concerning participants’ interactions with the platform, as shown in Figure 7, we see that the check-in of prevention activities was the foremost action performed by users in the system. This finding is important because, in the definition of the model, it was established that the performance of PI prevention activities is its primary objective. Nevertheless, it can be seen that among the preventive activities, skincare was the most performed, followed by body hydration and asepsis, i.e., the treatment given to the skin to prevent moisture or dryness is evidenced in these three activities.

Conceptually, the other practices: challenges, messages, risk assessment, and support material, are determined in the model as complements that aim to improve the interaction between prevention and the subjects. That said, it is possible to visualize in the graph that the most performed practice after the check-in was the messages stating people’s emotional state. Thus, it was seen the need for the wheelchair user to show how he was feeling, opening communication with his peers and making it possible to receive messages of support, reminders, thanks, or personalized messages that may contribute to reinforcing or mitigating the condition revealed. It was also observed that the use of media and risk assessment were resources used on a smaller scale. However, they had their role in complementing and ratifying the need for daily care to prevent PI.

Observing Table 5 about the points accumulated per practice, it is a fact that the distribution of the points was purposely thought out in the conception of the model, aiming to ensure that the check-in was the most crucial element of all the practices. It was noted that the users performed all the gamified collaborative practices offered to a greater or lesser frequency and that the subjects scored an average of 449 points in using the platform at the end of 7 days.

In this sense, it was possible to observe that gamification influenced the collaborative practices, inferring that gamification fulfilled its role of inserting game elements into the environment, allowing feelings to emerge such as enthusiasm, motivation, the spirit of competition, the satisfaction of feeling valued, commitment, the desire to want more, and the will to be better, that is, a playful environment was created in which participants had elements that helped them perform their activities. So far, we have demonstrated the subjects’ relationship with how gamification was perceived. Finally, we will present graphs containing the interactions between the volunteers when carrying out the collaborative practices.

As we can observe in Figure 8, which shows the messages between peers by weighted input and output grades, participants are close to each other, not needing many intermediaries to interact with other network elements. Ratifying this information, we have the network’s diameter calculated as 2, i.e., the most significant distance between the farthest nodes, in this graph, is maximum on two edges. It is important to note that during data modeling, a preference was identified for collective messages when all network members received the content shared by one sender. This trend showed that participants valued collaborative relationships more than individual ones, as recommended by the model.

Related to the behavior of individuals when it comes to identifying them as authorities or hubs in the network (Figure 9), it is important to argue that the fact that many hubs and authorities are identified shows that the messaging network is relatively distributed, i.e., it does not have pronounced dependencies in its communication flow.

The outgoing flows are equivalent to the realization of the practices. The incoming flows refer to the communication to the group of the concretization of these executions. In the graph, the values of the weighted input degrees form a shorter interval where all the nodes present slight variations, demonstrating that everyone participates in the activities in the environment. In the case of the outputs, the distance between the minimum and the maximum is more extensive, and it can be inferred that everyone involved participated in different intensities.

It is a fact that in the group experience, several roles are played by its members, in which some prefer to listen and those who want to talk, those who are willing to lead and those who choose to be led, those who want to participate and those who choose to observe. In this sense, Yalon and Leszcz, infer that “The need to belong is innate in all of us” [40] (p. 63), that is, belonging to a group that can accept and value its members is important. However, it is a process that takes time and is imbricated with the trust and belonging that lead to group cohesion.

Thus, regardless of the performance, participants must stay connected, and each one, in their own time, can contribute to achieving the purpose of this group, which is PI prevention. Thus, it is valid to highlight the volunteers’ statements at the end of the experiment evaluation, according to Table 7.

Thus, it was possible to identify the impression that the platform caused on the research subjects in a more individualized way. Subjects #2, #8, #9, and #10 concisely define the opinion that the application met the expectations and wishes of the users. Volunteers #1, #5, and #7 emphasize the need for improvements in the platform, especially regarding notifications and alerts to keep the connection between user and prevention always active.

It is important to note that subject #1 said that the platform is not very useful because he never had PI. When questioned about this sentence, the participant clarified that he already does these activities naturally in his daily life and that he would not need a platform to change his habits, since he is already adapted to this routine, but that he believes that for other people the platform is important in helping this change of habits.

Participant #4, in turn, emphasizes that the support network created in ColaborAtiva helps promote health and quality of life, which is precisely the idea we want to achieve with the model applied. Participant #3 approaches creativity in caring for wheelchair patients and how interesting the experience was, and #6 explains how easy it is to neglect prevention activities and how difficult it is to treat PI, and concludes that the platform serves as a warning for wheelchair users to know how they can prevent the development of PI.

Thus, collaborative practices evidenced in checking in on activities, sending messages between peers and also to sponsees, accessing informative materials suggested by therapists, performing risk assessments, encapsulated by a gamified system of points and challenges, and ended by a collaborative network connected through the actions performed during interactions on the platform had their results synthesized by the SUS instruments, UEQ, NPS, graphs, and observations ratifying that ColaborAtiva reflected the structures of the model created to prevent PI and highlighted that prevention through collaborative practices when gamified motivated wheelchair users to perform such actions.

After presenting the results and carrying out the necessary discussions, it was possible to answer the motivating question of this work based on instruments positive returns, explaining: the platform usability is at an acceptable level according to SUS; users, in general, rated the experience as positive according to UEQ; according to the platform promotion informed, it is considered that it is of quality; observations about gamification suggest that it encourages the use of the platform; and the interactions between wheelchair users generating a network represented by graphs. Thus, it is possible to assume that the structure adopted by the platform assists in PI prevention through engagement and collaboration.

It is worth stating that this research involved the volunteers, therapists, and researchers in the collaborative development of the platform, seeking feedback during building the software to ensure a result close to that expected by wheelchair users.

Finally, it is essential to create inclusive environments that allow disabled people to create relationships with other people who experience the same problems. And, by supporting each other, allow the strengthening of ties aiming at emotional support in creating the habit of promoting health and improving the quality of life.

## 5. Conclusions

The goal was to validate a platform based on a model of gamified collaborative practices that contributes to PI prevention through interaction among wheelchair users.

In this way, 16 wheelchair users evaluated the software, which obtained positive results in the evaluations with reservations for suggestions for modifications and improvements in usability and user experience that affected the application. However, it was unnecessary to modify the model to meet the requests. In addition, this work does not have absolute results and it is known that 16 wheelchair users do not represent the universe of existing subjects, but with this quantitative it was possible to observe a qualitative nature behaviors’ allowing to quantify the results for analysis and inference purposes, waiting in the next platform versions, carry out new tests with a greater volume of evaluators.

It is important to report the need to look at people who need daily health care. It should be assumed that it is possible to adapt the platform by applying it to situations requiring special care daily, such as diabetics, cardiac patients, the elderly, and smokers, among others.

Thus, future works include creating a new version with updates aiming to include the platform in the Play Store and Apple Store using the suggestions addressed by wheelchair users in their evaluations, for example, the use of smartphone notification system to receive platform users’ updates. Another project to be developed is constructing a software agent equipped with artificial intelligence capable of managing the collective and individual dynamics of ColaborAtiva users learning their routine and suggesting actions that benefit them about their health.

The integration of the platform with devices capable of providing data and information. Such as pillows and mattresses with sensors or wearable devices that provide body pressure against surfaces. For example, the using pads with sensors to identify pressure zones and help relieve these stress points by seeking a better posture [41] and automatically mark at check-in, on platform, the “appropriate positioning” item if the user can maintain the posture throughout the day. Another possibility is using the smartphone’s accelerometer to assist pressure relief maneuvers execution [42] and automatically mark at check-in, on platform, the “pressure relief maneuvers” item when finishing the activity.

As for the gamification aspect, it is possible to include short narratives to contextualize and involve the subjects from the perspective of health promotion and quality of life at a challenge.

Finally, all the proposed steps were rigorously followed, ensuring that the methodological path was followed, contributing to people having a better quality of life, and creating ramifications so that this work can evolve and serve the community.

## Figures and Tables

**Table 1:** Literature review.

**Stage 1**—search
Timespan: 2017 a 2022. Knowledge Bases: Periódicos Capes, PMC, SCIELO, Springerlink and SCOPUS. Descriptors: (“collaborative practices”) AND (gamification OR gamified)
Result: 55 documents (only Scopus presented results)
**Stage 2**—inclusion criteria (title, summary and keywords)
1. consider only original articles in journals (18 discarded); 2. consider only articles employing Collaborative Practices or Gamification (31 discarded);
Result: 6 documents.
**Stage 3**—inclusion criteria (full text)
1. consider only articles employing Collaborative Practices and Gamification (4 discarded);
Final Result: 2 documents.

**Table 2:** UEQ concept valuation example [28].

	1	2	3	4	5	6	7	
Original								Conventional

**Table 3:** ColaborAtiva platform screens.

Screen	Description
Registration and Login	Registers the name, nickname, e-mail that identifies the user, and the respective password. After this procedure, the participant receives an account’s activation e-mail, and after activating it, he/she can login.
Panel	Figure 3a presents all performed actions on the platform where everyone can access their realized activities feedback and from their peers through notifications system or participants messages.
Checkin	Figure 3b makes available the necessary participants’ activities that must be carried out daily to prevent PI.
Sending and Receiving Messages	Figure 3c provides communication between peers through previously registered or personalized messages.
Sponsorship System	Based on self-help groups, such as alcoholics anonymous (AA), a godfather/godmother becomes a mentor for one or more godchildren. Thus, bringing this concept to the platform when registering, the system chooses the participant’s godson to him/her take care of and assumes the commitment to pay more attention to him/her.
Risk assessment	It evaluates the risk of acquiring PI based on 6 subscales: sensory perception, humidity, activity, mobility, nutrition, and friction and shear. The subscales from 1 to 5, respectively, vary between 1 and 4 points, and the last one varies between 1 and 3. The lowest resulting between 6 and 9 points from subscales sum is the highest risk indicating a VERY HIGH RISK and the least worrisome level between 19 and 23 points representing NO RISK. Moreover, for comparison and follow-up, the last 5 evaluations are stored.
Materials	Supports participants sharing prepared or indicated media by therapists who follow the wheelchair users’ reality selecting texts, audios, or videos to improve the life’s quality of these people through communication.
Challenges	Individual or collective goals that, when met, are rewarded with stars and points. They are users’ incentives to induce them to stay active in the platform.
Scoreboard	Dailies and general ranking display the score after each practice is done. The points’ registration per day aims to encourage new participants since everyone has their points reset at the beginning of the day no matter what actions preceded the current date. However, on the general scoreboard, all the practices carried out are counted since the participant joined the platform.

**Table 4:** Volunteers profiles.

Gender (quantity)	Male 6	Female 10		
Age Groups (years) (quantity)	20–29 3	30–39 7	40–49 4	50–59 2
Smartphone Use per day (quantity)	less than 1 h 1	between 1 h–3 h 4	between 3 h–5 h 1	more than 5 h 10
Smartphone Manufacturer (quantity)	Samsung 9	Motorola 3	LG 3	Apple 1

**Table 5:** Points accumulated per practice.

Practice	Points
Performing the Checkin	6740
Sending Messages in General	198
Fulfillment of Challenges	130
Sending Messages from the Godfather or Godmother to the godchild	48
Access to Information	45
Performance Risk Assessment to prevent PI development	28

**Table 6:** Observations about gamification.

Subject	Observations
#1	I was actually excited because it was the second day, and overall I was eighth or ninth.
#2	The first few days I worked hard and got a good score.
#3	Influences a little on the motivation to do the activities
#4	I looked at the scoreboard, and I was in 3rd, but I do not remember the exact score. Furthermore, that influenced me to do the activities.
#5	The last time I looked, I was among the first, some days I was first, some days I was second. The ranking did not influence much, but I know that it gives you a little itch that is like an affirmation that you are doing well, that you are taking the purpose seriously
#6	Having a significant score does influence it because you end up competing indirectly with the other participants, and it also ends up influencing you to want to score more.
#7	When I looked at the ranking and saw that it was low, I wanted to improve.
#8–#16	Made no observations.

**Table 7:** Post-experimental observations.

Subject	Remarks
#1	As I do not have bedsores or related problems, the app could not be useful. I congratulate, however, on the initiative and hope that the application can undergo the necessary changes to become more pleasant and interesting to the target audience.
#2	The platform completely met my expectations.
#3	The elaboration of the platform is very interesting. I know it will help other people as much as it helped me. Thank you for the opportunity to participate. I am immensely grateful for thinking interestingly and creatively of caring for people with disabilities who go through these uncomfortable and undesirable situations. Congratulations on your initiative and care for others.
#4	Collaboration and support network promotes health and quality of life, this platform favors this.
#5	I believe that with a few improvements, this platform can help many people. Thank you for giving me this unique experience.
#6	The platform in question is very relevant to make routine activities, which are often forgotten, as important in the life of the person with a disability, so that they can pay attention and police themselves of measures that can prevent pressure injuries. I, specifically, have had at least 4 (four) PI, one of which I had to have surgery on the ischial region because it would not close. However, this bedsore served to redouble my care with pressure ulcers, such as, for example, buying a Roho cushion, as well as performing pressure relieves. In this way, this platform could, can, and will be of extreme importance to alert the person, whether disabled or not, to take the proper care of their health and, thus, avoid pressure injuries. Finally, I wish success to the team on behalf of the researcher. Thank you very much!
#7	I really believe in the platform, but it cannot disconnect every time you leave it for another one. It could issue alerts to remind you of activities or when a message is directed.
#8	I liked it a lot.
#9	Great platform.
#10	All great.
#11–#16	Made no observations.

**Table 8:** Smartphone usage habits by age group in Canada (2018) [37].

Questions	Age Group (Years)			
	15–24	25–44	45–64	65+
Do you have a smartphone?	97.9%	97.1%	87.0%	60.4%
Do you check your smartphone at least every 30 min?	57.5%	51.9%	39.5%	21.2%
Before you go to sleep, the last thing you do is check your smartphone.	72.6%	64.9%	45.4%	30.7%
You use your smartphone while watching television.	64.6%	64.3%	44.4%	25.7%
Use your smartphone during dinner.	35.8%	24.8%	12.5%	6.0%
